# COX-2 Inhibition by Bioactive Peptides from Peanut Worm (*Siphonosoma australe*) Collagen Through *in vitro* Digestion Simulation

**DOI:** 10.17113/ftb.63.03.25.8691

**Published:** 2025-08-31

**Authors:** Suwarjoyowirayatno, Chusnul Hidayat, Tutik Dwi Wahyuningsih, Retno Indrati

**Affiliations:** 1 Department of Food and Agricultural Product Technology, Faculty of Agricultural Technology, Universitas Gadjah Mada, Flora Street, Bulaksumur, Yogyakarta 55281, Indonesia; 2 Department of Fish Product Technology, Faculty of Fisheries and Marine Science, Universitas Halu Oleo, H.E.A. Mokodompit Street, Kendari 93231, Indonesia; 3 Department of Chemistry, Faculty of Mathematics and Natural Sciences, Universitas Gadjah Mada, Sekip Utara, Bulaksumur, Yogyakarta 55281, Indonesia

**Keywords:** bioactive peptide, COX-2 inhibition, digestion simulation, molecular docking, peanut worm collagen, *Siphonosoma australe*

## Abstract

**Research background:**

Chronic, unregulated inflammation is a crucial factor in the development of numerous diseases and is closely linked to the increased expression of cyclooxygenase-2 (COX-2). While various bioactive peptides from marine organisms have shown COX-2 inhibitory effects, peptides derived from the collagen of the peanut worm (*Siphonosoma australe*) have not yet been demonstrated. The aim of this study is to investigate the potential COX-2 inhibitory activity of peanut worm collagen by simulated digestion *in vitro* with pepsin-pancreatin followed by molecular docking.

**Experimental approach:**

During simulated *in vitro* digestion, commercial pepsin (at pH=3) and pancreatin (at pH=7.5) were applied for 240 min at 37 °C to evaluate the degree of hydrolysis, peptide concentration and COX-2 inhibitory activity. The samples with the most significant COX-2 inhibitory activity were then separated into fractions and identified.

**Results and conclusions:**

The 210-minute simulated digestion *in vitro* showed the highest COX-2 inhibitory activity (64.31 %). This result was confirmed by the increased degree of hydrolysis (DH) and peptide concentrations observed during the simulated *in vitro* digestion. The peptide fraction of <1 kDa had the highest inhibitory activity (89.05 %) and was subsequently subjected to sequencing analysis. Three novel peptides, ADIAGQAAQVLR, LNNEITTLR and VGTVEK, were identified and confirmed to contain crucial amino acids and therefore verified as COX-2 inhibitors. VGTVEK has the most potent interaction, as shown by the lowest binding energy (−4.41 kcal/mol). The molecular docking revealed that VGTVEK (631.35 Da) binds to the active site of COX-2 and forms hydrogen bonds with Gln178, Leu338, Ser339, Tyr371, Ile503, Phe504, Val509 and Ser516 and hydrophobic interactions with Met99, Val102, Val330, Ile331, Tyr334, Val335, Leu345, Trp373, Leu517 and Leu520. Other biological activities of the produced peptides included ACE inhibitors, dipeptidyl peptidase-IV (DPP-IV) inhibitors and α-glucosidase inhibitors. After toxicity prediction, the peptides were classified as non-toxic.

**Novelty and scientific contribution:**

The study found that peptides derived from peanut worm collagen have the potential to be novel, natural agents for anti-inflammatory therapy. Their broader application in functional foods, nutraceuticals and pharmaceuticals could provide new options for people suffering from inflammation and support both treatment and maintenance of overall health.

## INTRODUCTION

Cyclooxygenase-2 (COX-2) is crucial for the conversion of arachidonic acid to prostaglandins, which are key regulators in inflammation. The activation of pro-inflammatory mediators triggers the expression of COX-2. The upregulation and overexpression of COX-2 are primarily associated with inflammatory processes (*1*). Inflammation is a host's defence mechanism that responds adaptively to injury, tissue damage and infections. Although inflammation generally has protective benefits for the body, persistent and unregulated inflammation can play a role in the development of numerous diseases (*2*). It is the starting point for numerous chronic diseases, such as asthma, cancer, skin diseases, cardiovascular diseases, neurological disorders and arthritis. Inflammation is usually treated with non-specific small-molecule drugs (*3*). Although various anti-inflammatory medications are commercially available, they are all associated with potential side effects (*4*). These drugs are frequently associated with gastrointestinal (GI) side effects, including peptic ulcers, GI bleeding, intestinal obstruction and GI erosion (*5*).

Concerns about the adverse effects of synthetic substances have increased interest in the use of natural compounds and their derivatives as safer alternatives for therapeutic purposes, including functional foods and nutraceuticals (*4*). Numerous efforts are focused on the development of alternative and more selective anti-inflammatory treatments, some of which include peptides. Peptides have emerged as the preferred compounds for various targets due to their high specificity and the application of recent and innovative synthetic methods (*3*). Anti-inflammatory peptides are present in many living organisms, with numerous peptides from plants, mammals, bacteria and marine organisms demonstrating remarkable anti-inflammatory properties (*6*). Anti-inflammatory peptides have recently been identified and extracted from specific species of peanut worms. Specifically, peptides derived from *Sipunculus nudus* show anti-inflammatory activity through multiple mechanisms, including reduction in the expression of pro-inflammatory mediators and inhibition of COX-2 (*7*, *8*).

Bioactive peptides, usually short chains of amino acids derived from proteins, show a range of biological activities beyond their nutritional value. These peptides are normally inactive in the context of their parent proteins and require proteolytic cleavage to be released and exert their specific bioactive functions (*4*). Proteolytic processes include food processing, microbial fermentation, germination and the activity of various protease enzymes (*9*). Anti-inflammatory peptides have been identified in peanut worms that use proteolytic enzymes for hydrolysis (*2*). Digestive enzymes have the ability to degrade peptides, leading to changes in their bioactivity in the gastrointestinal tract. Enzymatic hydrolysis during food processing can produce peptides with shorter amino acid chains (*10*). Hydrophobic amino acids in short-chain peptides are associated with biological activities that confer health benefits (*9*).

Anti-inflammatory peptides derived from food generally contain between 2 and 20 amino acids. Apart from their length, the composition and sequence of these peptides are essential for their anti-inflammatory effect (*11*). Recently, Sangtanoo *et al.* (*8*) isolated two peptides, LSPLLAAH (821.48 Da) and TVNLAYY (843.42 Da), from the peanut worm *Sipunculus nudus* after hydrolysis with Alcalase, Neutrase and Flavoursome enzymes. These peptides showed strong anti-inflammatory effects in lipopolysaccharide-stimulated RAW264.7 macrophages and significantly reduced the expression of pro-inflammatory mediators (iNOS, IL-6, TNF-α and COX-2) after 12 h of treatment. Conversely, Lin *et al.* (*7*) extracted peptides from *S. nudus* collagen using hydrolytic proteases derived from animals and flavour proteases. The study showed that these peptides alleviated inflammation in mouse skin wounds by reducing the mRNA expression levels of TNF-α, TGF-β1 and IL-1β.

The amino acid sequence of low-molecular-mass peptides produced during the simulated digestion of peanut worm collagen could be a promising source of bioactive peptides with COX-2 inhibitory properties. In Indonesia, *Siphonosoma australe* is rarely consumed as food, while in China, it is mainly used as a raw material in traditional medicine (*12*). Compared to other marine organisms such as fish, molluscs and crustaceans, *S. australe* is still scarcely studied scientifically, especially its anti-inflammatory potential. *S. australe* is found exclusively in the waters of eastern Indonesia and Australia (*13*) and has a a characteristic protein and amino acid composition. Consequently, there is a great opportunity to identify novel bioactive peptides that could contribute to further development of alternative natural compounds and nutraceuticals in the formation of anti-inflammatory medications. The amino acid composition of *S. australe*, which contains both positively charged and hydrophobic amino acids, contributes to its anti-inflammatory properties (*14*). In our previous research, *Siphonosoma australe* collagen was found to inhibit COX-2 by 41.12 %, with an IC_50_ value of 59.9 μg/mL (*15*). The anti-inflammatory activity of *S. australe* can be further enhanced by enzymatic hydrolysis, facilitating the production of more potent COX-2 inhibitory peptides. However, there are no published studies on the use of *S. australe* as a collagen source for the production of COX-2 inhibitory peptides. Therefore, the aim of this study is to collect thorough information about the profile of COX-2 inhibitory peptides in *S. australe* by conducting simulated *in vitro* digestion with pepsin and pancreatin. The hydrolysate with the most significant COX-2 inhibitory potency was fractionated with a molecular mass cut-off (MMCO) membrane, analysed for its inhibitory activity and subjected to molecular docking for characterisation.

## MATERIALS AND METHODS

### Materials

Peanut worms (*S. australe*) were sourced from Rombo Village in Southeast Sulawesi Province, Indonesia, in dried form. The *o*-phthaldialdehyde (OPA) was obtained from Merck, Rahway, NJ, USA. Pepsin (EC.3.4.23.1 from porcine gastric mucosa P7012), pancreatin (EC.232-468-9 from porcine pancreas P7545) and the COX-2 inhibitor screening kit (fluorometric) (MAK399) were obtained from Sigma-Aldrich, Merck, St. Louis, MO, USA. Regenerated cellulose membranes (Membra-Cel) with molecular mass of 1, 3.5 and 14 kDa (MD44) were acquired from Viskase Co., Lombard, IL, USA. All other chemicals were of analytical grade.

### Preparation of peanut worm collagen

The collagen extraction was modified by Chuaychan *et al.* (*16*). In order to remove non-collagen proteins, peanut worms were immersed in 0.1 M NaOH at 4 °C for 6 h at a 1:10 ratio (10 %), changing the solution every 3 h. For demineralisation, the worms were treated with 0.5 M disodium EDTA at a pH=7.4 for 48 h at the same 1:10 ratio, with the solution being replaced every 24 h. After demineralisation, the worms were incubated in 0.5 M CH_3_COOH (1:10) for 48 to 72 h at 4 °C with agitation. The solution was filtered through a double layer of cheesecloth. NaCl was then added to the collagen solution until a final concentration of 2.6 M was reached, resulting in collagen precipitation. The precipitate was collected after centrifugation at

15 000×*g* and 4 °C for 30 min and subjected to dialysis using a 14 kDa molecular mass cut-off (MMCO) membrane in 0.1 M CH_3_COOH (volume ratio 1:30) for 48 h at 4 °C. The resulting solution was freeze-dried to obtain acid-soluble collagen from the body wall of peanut worm.

### In vitro simulated digestion

An *in vitro* digestion method was used in this study, following the procedure described by Puspitojati *et al.* (*17*), which was slightly modified. Each sample was adjusted to pH=3 using phosphate-buffered saline (Oxoid, Basingstoke, UK) and 1 M HCl, achieving a protein concentration of 5 mg/mL. Pepsin (EC 3.4.23.1, 2,000 U/mL) was added, maintaining an enzyme-to-substrate ratio of 1:10. The reactions were carried out at 37 °C for 120 min, with samples being taken at 30-minute intervals for analysis. After the 120-minute pepsin digestion, the hydrolysates were subjected to a duodenal digestion simulation. The pH was increased to 7.5 by adding 2 M NaOH solution, followed by the addition of pancreatin (EC 3.4.21.1, 100 U/mL) at an enzyme-to-substrate ratio of 1:25. The reactions were incubated at 37 °C for another 120 min, with samples taken for analysis every 30 min. The hydrolysis was terminated by heating the solution to 100 °C for 10 min and adjusting the pH to 7 with 2 M NaOH. The hydrolysate was centrifuged at 8000×*g* and 4 °C for 10 min and the supernatant was stored at −20 °C for future use.

### Peptide fractionation using MMCO

The peptides were fractionated using a membrane with an MMCO of 1, 3.5 and 14 kDa, following the protocol described by Agustia *et al.* (*18*). Before use, the MMCO membranes were activated with 10 mM NaHCO_3_ and 10 mM Na_2_EDTA solutions. To rehydrate the membranes, they were immersed in NaHCO_3_ solution for 10 min and then transferred to Na_2_EDTA solution for further 10 min. The membranes were subsequently placed back into a fresh Na_2_EDTA solution and boiled for another 10 min. After the activation, the membranes were thoroughly washed with sterile distilled water and were deemed ready for use.

Peptide solutions with a protein concentration of 10 mg/mL were filtered through a 1 kDa membrane, ensuring that both sides were securely sealed with a membrane clip. The membranes were then placed in Aqua Pro water (Axios Pharmindo, Jakarta, Indonesia) in a beaker and maintained at a stirring speed of 80-100 rpm with a magnetised stirring rod. The fractionation was carried out at 4 °C for 12 h. After this period, the 1-kDa peptide fraction was separated using Aqua Pro solution. The remaining solution was sequentially filtered through membranes with MMCOs of 3.5 and 14 kDa, following the same procedure. The resulting fractions contained peptides with molecular masses of less than 1 kDa, between 1 and 3.5 kDa, 3.5 and 14 kDa, and greater than 14 kDa. These peptide fractions were stored at -20 °C until further analysis.

### Determination of the peptide concentration

In this study, the peptide concentration was determined using a method based on the investigations of Church *et al.* (*19*). The OPA reagent was prepared by mixing 25 mL of 100 mM Na_2_B_4_O_7_, 2.5 mL of 20 % NaC_12_H_25_SO_4_ and 1.1 mL OPA (40 mg OPA dissolved in 1 mL CH_3_OH and 100 mL C_2_H_6_OS, followed by the addition of 21.4 mL distilled water. The sample (20 μL) was added to 1 mL of the prepared OPA reagent, incubated at room temperature for 2 min and then the absorbance was measured at 340 nm. The peptide concentration was determined using Tryptone as a reference standard and a standard linear regression curve was constructed with a concentration range from 0 to 1.50 mg/mL (0, 0.25, 0.50, 0.75, 1.0, 1.25 and 1.50 mg/mL).

### Determination of the degree of hydrolysis

The degree of hydrolysis (DH) was determined using a modified method according to Lin *et al*. (*20*). The collagen from peanut worms was hydrolysed with an 8 M hydrochloric acid solution (1:10 ratio) and incubated at 110 °C for 24 h. After neutralisation with 8 M NaOH solution, the mixture was adjusted to a final volume of 10 mL with distilled water and filtered. The peptide concentration was then measured with the OPA reagent. The degree of hydrolysis (%) was determined using the following formula:


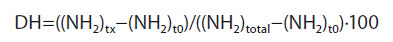
 /1/

where (NH_2_)_tx_ is the amount of free amino acids at x minutes, (NH_2_)_t0_ is the amount of free amino acids at 0 minutes and (NH_2_)_total_ is the total amount of free amino acids present.

### Determination of COX-2 inhibition

The inhibition of COX-2 enzymes by collagen *in vitro* was investigated using the fluorometric COX-2 inhibitor screening kit from Merck (Darmstadt, Germany). The assay was conducted in a 96-well plate, with a mixture of 10 μL arachidonic acid/NaOH solution, 1 μL recombinant COX-2, 76 μL COX assay buffer, 2 μL COX-2 cofactor working solution, 10 μL test sample and 1 μL COX probe solution. Fluorescence kinetics was measured at 25 °C for 10 min using a Spark® multimode microplate reader (Tecan, Männedorf, Switzerland) with excitation and emission wavelengths set to 535 nm and 587 nm, respectively. Celecoxib served as a positive control, while the negative control consisted of the enzyme without any drugs or peptides. All peptide and control samples were tested in triplicate and the reaction conditions followed the standard protocol provided with the Merck COX-2 inhibitor assay kit.

To obtain the corresponding fluorescence values (relative fluorescence unit - RFU1 and RFU2), two points (T1 and T2) were selected within the linear section of the graph. The slope (S) for each sample was calculated by dividing the net fluorescence change (∆RFU) by the corresponding time interval (∆T) according to this formula:


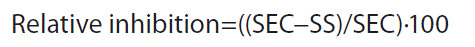
 /2/

where SEC is the slope of enzyme control and SS is the slope of the sample.

### Characterisation and identification of peptide sequence

The peptide fraction that exhibited the highest COX-2 inhibitory activity after digestion simulation was analysed and identified using liquid chromatography (Thermo Scientific™ Vanquish™ UHPLC Binary Pump) combined with Orbitrap high-resolution mass spectrometry (Thermo Scientific™ Q Exactive™ Hybrid Quadrupole-Orbitrap™ high-resolution mass spectrometer) (Waltham, MA, USA) (*21*). Peptides were separated using gradient elution with a mobile phase consisting of MS-grade water with 0.1 % formic acid (A) and MS-grade acetonitrile with 0.1 % formic acid (B). The gradient was set as follows: 0–1 min at 5 % B, a linear increase from 5 to 50 % B for 1–31 min, followed by 2 min at 50 % B. The initial conditions were restored and held for 47 min. The flow rate was maintained at 0.075 mL/min and a C18 HPLC column (150 mm length, 1 mm i.d., 3 μm particle size; Thermo Scientific™ Acclaim™ PepMap™) with a 3 μL injection volume was used. Mass spectrometry was analysed using positive ionization in full MS/dd-MS2 mode. The conditions included a sheath gas flow rate of 15 AU, an auxiliary gas flow rate of 5 AU, a capillary temperature of 300 °C and a spray voltage of 4.00 kV. The scan range was 150–2000 *m*/*z* with resolutions of 140 000 for full MS and 17 500 for dd-MS2. Peptide sequences were identified using Proteome Discoverer v. 2.2 (*22*, *23*) and MaxQuant v. 2.4.14.0 software (*23*). The identified peptide sequence was compared for similarity using https://www.uniprot.org/, accessed on 28 February 2024 (*16*).

### Biological activity of peptide sequence evaluation

Following the method described by Minkiewicz *et al.* (*24*), BIOPEP-UWM was used to determine the potential biological activities of the fractionated peptides (https://biochemia.uwm.edu.pl/biopep-uwm/, accessed on 6 March 2024. This database provides A and B values, which represent the frequency of bioactive fragments and the biological activity of protein fragments, respectively, and allow characterisation of the biological properties of the sequence. Additionally, the toxicity of the peptide sequences was evaluated using the ToxinPred database (*25*) (https://webs.iiitd.edu.in/raghava/toxinpred/, accessed on 1 March 2024).

### Molecule preparation for computational modelling

The three-dimensional (3D) structure of the peptide was created using novoprolab.com. First, the peptide sequence was converted into the SMILES string format (*26*) (https://www.novoprolabs.com/tools/convert-peptide-to-smiles-string, accessed on 6 March 2024). Then, the SMILES string was used to create a 3D structure (https://www.novoprolabs.com/tools/smiles2pdb, accessed on 6 March 2024) (*26*). The 3D structure was optimised by minimising its energy with Yasara structure software (*27*). Moreover, the three-dimensional configuration of the COX-2 enzyme was obtained from the protein data bank (https://www.rcsb.org/) under PDB ID 3LN1 (*28*). The structure of the enzyme was refined by eliminating water and irrelevant ligands through Yasara structure software (*27*).

### Molecular docking of the peptide and COX-2

The docking was carried out with the Yasara structure application, using a force field scoring functional approach to compute the binding energy values (*27*). In this process, the docking macro command (dock_run.mrc) in Yasara structure was used. A cubical box with a 5.0 Å radius was centred around the native ligand to direct the docking. After the docking was completed, the results were stored in PDB (.pdb) file format. The post-docking data were then analysed and visualised using Discovery Studio Visualisation software (*18*).

### Statistical analysis

The experimental data are reported as mean values with standard deviations from three replicates. Before analysis, the data were tested for normality using the Shapiro-Wilk test. A one-way analysis of variance (ANOVA) was then conducted, followed by Duncan's multiple range test (DMRT) for comparative analysis. A *t*-test was used to evaluate significant differences between the mean values of the two groups of hydrolysate peptides. All statistical analyses were carried out using SPSS IBM v. 25 (IBM, Armonk, NY, USA) with a significance threshold set at 5 % (*18*).

## RESULTS AND DISCUSSION

### Degree of hydrolysis

The hydrolysis of peanut worm (*Siphonosoma australe*) collagen using pepsin and pancreatin reflects two critical stages in the human digestive process. Pepsin acts in the gastric phase, while pancreatin functions in the small intestinal phase (*29*). The concentrations of these enzymes are consistent with their reported activity levels in the stomach and intestine (*30*) and ensure effective collagen hydrolysis to produce bioactive peptides with potential anti-inflammatory effects. Sampling at 30-minute intervals reflects the typical duration of gastric and intestinal digestion (*29*) and allows tracking of peptide formation at different stages (early, middle and late stages of digestion). The peptide samples will provide an in-depth profile of peptide release and degradation over time and allow the identification of optimal hydrolysis stages at which the peptides exhibit maximum bioactivity.

**Fig. 1:**
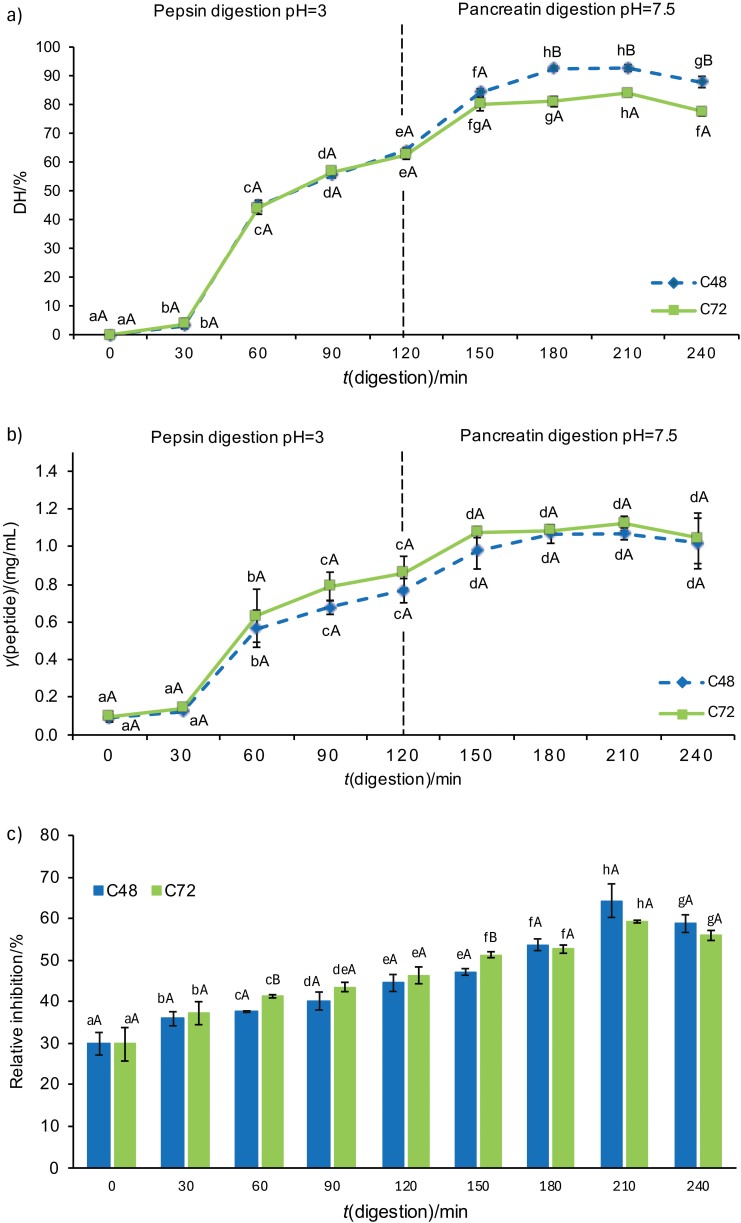
The results for: a) degree of hydrolysis, b) peptide concentration, and c) COX-2 inhibitory activity of peanut worm collagen during 240 min of digestion simulation with pepsin and pancreatin. Mean value±S.D., *N*=3. Values marked with different lowercase letters are significantly different according to Duncan’s test (p<0.05) and those with capital letters are significantly different according to *t*-test (p<0.05). C48 and C72=hydrolysates obtained from peanut worm collagen during 48 and 72 h of extraction, respectively

The hydrolysis patterns observed in the peanut worm collagen extracts during 48 (C48) and 72 h (C72) were similar (Fig. 1a). The degree of hydrolysis (DH) values for both samples increased from 0 to 120 min with pepsin, followed by an increase in hydrolysis from 120 to 240 min after the addition of pancreatin. The DH peaked at 210 min, with C48 showing a value of 92.71 % and C72 showing a value of 84.03 % (p<0.05). The higher DH observed in the 48-hour collagen extraction can be attributed to the production of longer collagen polypeptide chains than in the 72-hour extraction. The shortening of collagen chains by acetic acid is more pronounced after 72 than after 48 h. As a result, the collagen polypeptide chains remain longer after 48 h and provide more substrates for cleavage by the pepsin-pancreatin enzymes. Due to the different substrate specificities, the cleavage patterns of pepsin and pancreatin improve the efficiency of the hydrolysis process (*10*). The specificity of each enzyme results in the more effective and efficient production of smaller peptides. The study found that the DH was higher when pancreatin was applied to peanut worm collagen than pepsin. This result is consistent with that of Agustia *et al.* (*18*), who reported lower hydrolysis of jack bean protein with pepsin than with pancreatin. In addition, pancreatin led to a greater increase in hydrolysis than pepsin (*31*).

### Peptide concentration

The peptide concentration values followed a pattern similar to the degree of hydrolysis (DH). After 240 min of hydrolysis with pepsin and pancreatin, the higher DH resulted in increased production of peptides, with the peak concentrations reaching 1.07 mg/mL for C48 and 1.12 mg/mL for C72 (Fig. 1b). The application of pepsin for 0-120 min effectively increased peptide concentration, followed by the addition of pancreatin from 120 to 150 min, which significantly increased peptide concentration (p<0.05). Pepsin specifically cleaves the telopeptide bonds in collagen, producing smaller peptides with a strong specificity for hydrophobic amino acids (*32*). Both endoprotease and exoprotease enzymes in pancreatin significantly enhance protein hydrolysis (*33*). The combination of pepsin and pancreatin effectively increases the yield of collagen peptides. These results suggest that pepsin is pivotal in breaking down the native, well-folded protein, while pancreatic enzymes generate shorter peptides (*34*).

However, between 150 and 240 min, the DMRT test for C48 and C72 revealed no significant difference (p>0.05). This can be attributed to a reduced peptide concentration after 240 min, suggesting a reduced availability of collagen telopeptide substrate for further hydrolysis (Fig. 1b). The observed decrease in the value after 240 min could be due to the continued hydrolysis of collagen peptides by the pancreatin enzyme, which produces free amino acids. As a result, the peptide chains shorten and the molecular mass distribution decreases as the DH increases. This ultimately leads to the cleavage of the peptide bonds and a subsequent increase in the concentration of free amino acid. This study is consistent with the findings of Khushairay *et al.* (*35*), who reported that the peptide content of the swallow’s nest hydrolysate treated with pancreatin increased during the first 90 mi of hydrolysis, but decreased after that. After an initial phase of rapid hydrolysis, the rate of hydrolysis typically slows down and eventually reaches a stationary phase. Although more cleavage sites are available, the rate of hydrolysis is influenced by the cleavage specificity of the enzyme and the accessibility of peptide bonds to the enzymes involved.

### COX-2 Inhibitory activity

The increase in DH and peptide concentration is closely related to the efficacy of the resulting COX-2 inhibitory activity. The COX-2 inhibition capacity of collagen hydrolysate was found to significantly increased as the increasing duration of hydrolysis (p<0.05) (Fig. 1c). Collagen samples C48 and C72 reached peak COX-2 inhibition at 210 min, achieving 64.31 and 59.27 %, respectively. This effect is attributed to the extraction of collagen with acetic acid followed by enzymatic digestion by pepsin and pancreatin, which produces small collagen peptides, as reflected in the peptide concentration and DH values. These peptides, which consist of hydrophobic and positively charged amino acids, can bind to the active site of COX-2 and inhibit its function. Anti-inflammatory peptides with COX-2 inhibitory activity usually contain hydrophobic amino acid residues such as alanine (Ala), glycine (Gly), tryptophan (Trp), valine (Val), tyrosine (Tyr), phenylalanine (Phe), isoleucine (Ile) and methionine (Met), alongside positively charged residues like arginine (Arg) and lysine (Lys) (*11*). The hydrolysed *S. australe* collagen inhibited the enzyme by 41.12 %, which exceeded that of its non-hydrolysed form (*15*). Additionally, the novel COX-2 inhibitory peptides YCS, YAD, WCD and GYW were detected with inhibition values of ≥27, ≥33, ≥35 and ≥45 %, respectively (*36*). The results are consistent with peptides WGD, WYS and WAY, which showed inhibition of ≥63, ≥67 and ≥68 %, respectively (*36*), and are similar to the short peptide H_2_N-Gly-Gly-Phe-Leu-OMe, which achieved 60 % inhibition (*37*).

Moreover, the COX-2 inhibitory activity in collagen samples C48 and C72 decreased after 240 min. This decrease is attributed to continuous hydrolysis during the 240-minute period, which degrades the peptides to free amino acids, possibly altering the amino acid sequences and decreasing or even cancelling the anti-inflammatory activity of the peptides. These results are consistent with those of Agustia *et al.* (*18*), who reported a decrease in DPP-IV inhibitory activity of jack bean peptides after 210 and 240 min of digestion. This decrease in activity is attributed to the specific sequence and composition of these peptides, which impair their ability to bind effectively to the enzyme and thus reduce their inhibitory potential.

### Peptide fractionation with MMCO

Peptides derived from simulated *in vitro* digestion were separated by membrane dialysis to investigate their molecular mass distribution and influence on COX-2 inhibitory activity. The use of different MMCO membrane sizes aims to optimise the identification of peptides with suitable sizes for bioactivity and therapeutic applications. Larger peptides tend to be more stable, while smaller peptides have higher biological activity and can permeate cell membranes better (*38*); consequently, the molecular mass of the peptide significantly influences the functional activity. Numerous studies have shown that different MMCO membrane sizes can yield peptides with different anti-inflammatory properties (*8*).

After 210 min of digestion, samples C48 and C72 were fractionated into four molecular mass categories based on MMCO: <1, 1–3.5, 3.5–14 and >14 kDa (Table 1). Among these fractions, peptides with a molecular mass below 1 kDa had the strongest COX-2 inhibitory activity (p<0.05), suggesting that smaller collagen peptides have a higher binding affinity for the COX-2 active site. The MMCO membrane facilitates the selective separation of low-molecular-mass peptides, thus enhancing the anti-inflammatory activity (*8*). These results suggest that collagen peptides with low molecular mass that inhibit COX-2 can be effectively produced by pepsin and pancreatin hydrolysis. The obtained results were higher than those of the peptides WAY, WCY and FCS, which had inhibition rates of ≥68, ≥70 and ≥78 %, respectively (*36*). Additionally, these results are similar to those for WCS peptide ≥88 % (*36*) and new selective COX-2 inhibitors (pyrimidine-5-carbonitrile) at 9.5 % (*39*). The results of this study are consistent with those of Rizkaprilisa *et al.* (*31*), who reported that tempe peptides subjected to hydrolysis by pepsin and pancreatin during digestion yielded an increased amount of low-molecular-mass peptides. Previous studies have shown that small peptide fractions, including those with molecular mass of <5, <3, 1, 0.8, 0.6 and 0.3 kDa, show significant anti-inflammatory effects by inhibiting the COX-2 enzyme (*7*, *8*). Additionally, the hydrolysis process under simulated *in vitro* gastrointestinal conditions promotes the formation of low-molecular-mass peptides, which can further enhance the COX-2 inhibitory activity.

**Table 1:** Inhibition of COX-2 activity with different peanut worm collagen hydrolysate fractions

*M*(fraction)/kDa	COX-2 inhibition/%	
	C48	C72
<1	(85.6±1.2)^dA^	(89.0±1.5)^dB^
1–3.5	(75.8±0.6)^cA^	(80.4±1.9)^cB^
3.5–14	(71.7±0.8)^bA^	(73.0±1.5)^bA^
>14	(62.0±2.5)^aA^	(63.3±1.8)^aA^

Mean value±S.D., *N*=3. The values with different lower-case letters in superscript in the same column are significantly different according to Duncan’s test (p<0.05), and the values with different capital letters in superscript in the same row are significantly different according to *t*-test (p<0.05). C48 and C72=hydrolysates obtained from peanut worm collagen during 48 and 72 h of extraction, respectively

Low-molecular-mass peptides are only recognised by enzymes to a limited extent and have fewer cleavage sites. This reduced enzymatic degradation enables these peptides to remain intact when they enter the bloodstream, allowing them to reach their target organs effectively (*40*). Unlike larger protein molecules and even smaller amino acid molecules, these peptides are efficiently absorbed by the body through specialised transport mechanisms in the intestine. They are then delivered to the target area to exhibit anti-inflammatory effects (*41*). Their transit mechanism minimises energy expenditure and reduces strain on the gastrointestinal system, which is particularly beneficial for people with chronic diseases (*14*). Once digested in the gut, low-molecular-mass peptides have been shown to have a positive effect on conditions such as arthritis (*42*) and breast cancer (*43*). Other concerns regarding small peptides are the number of peptides produced and their purity levels. Additional research is needed to evaluate the stability of these peptides against proteolytic enzymes and environmental factors including temperature, moisture activity, freeze–thaw cycles and storage time. Additionally, the absorption through the digestive tract and peptide-related parameters should be investigated by *in vivo* testing.

### Identification of peptide sequence of peanut worm collagen and its potential inhibitory effect

**Fig. 2:**
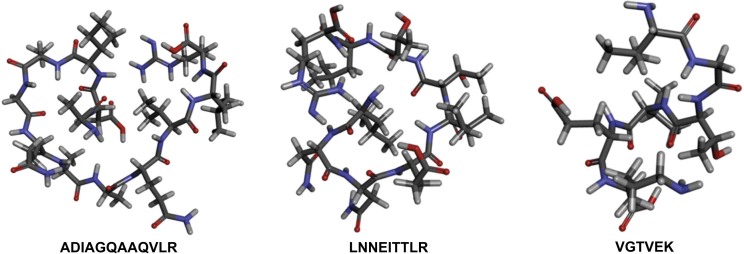
The 3D structures of peanut worm peptides

The peptide fractions that show the strongest COX-2 inhibitory activity (<1 kDa) from MMCO dialysis were sequenced. Three novel peptides with COX-2 inhibitory potential were identified: ADIAGQAAQVLR, LNNEITTLR and VGTVEK. The 3D structures of these peptides are shown in Fig. 2. The peptides had a molecular mass from 631.35 to 1212.37 Da. The presence of peptides larger than the membrane pore size can be attributed to variations in pore stability during the dialysis process and the molecular properties of the peptides. Additionally, peptides with molecular mass similar to the MMCO pore size can still pass through the membrane as their dialysis rates are influenced by factors such as molecular shape and solubility (*44*). Furthermore, the transport of peptides through membrane filtration is influenced by their charge, with peptide separation primarily resulting from the combined effects of the charge selectivity of electrodialysis and the size exclusion capability of the filtration membranes (*45*). Each peptide molecule has different properties, and factors such as concentration, interactions and hydrophobicity significantly affect its ability to diffuse through a dialysis membrane. It is crucial to consider variables such as temperature, volume, agitation and the frequency of external buffer exchange during the process (*44*). Despite exceeding the filter size, the peptide derived from the peanut worm falls within the range of inhibitory COX-2 peptides as specified in the BIOPEP-UWM database (955–1400 Da) (*24*).

The sequencing lengths of the COX-2 inhibitor peptide fragments varied from 6 to 12 amino acids. The N-terminal region of the peptide contains hydrophobic amino acids such as alanine (Ala), leucine (Leu) and valine (Val). In contrast, the C-terminal region contains positively charged amino acids, including arginine (Arg) and lysine (Lys). The presence of hydrophobic residues at the N-terminus and positively charged residues at the C-terminus increases the ability of the peptide to inhibit COX-2, with IC_50_ values ranging from (4.31±0.99) to (15.53±1.78) µg/mL (*46*). Singh *et al.* (*37*) found that the hydrophobic nature of the COX-2 active site led to the selection of Gly, Ala, Val, Leu and Phe for the synthesis of the COX-2 inhibitor peptide. Additionally, Hong *et al.* (*47*) demonstrated that tetrapeptides derived from walnut dregs, which contain hydrophobic amino acid residues at the N-terminus (LFPD, FPGA, AGFP and VGFP), formed strong hydrogen bonds and hydrophobic interactions with residues in the COX-2 active site.

The values of frequency of bioactive fragments (A) and possible biological activity of protein fragments (B) (Table 2) were derived from peptide sequence simulations using the BIOPEP-UWM database (*24*) and ToxinPred (*25*) to assess the potential toxicity of the peptides. According to the BIOPEP-UWM simulation, the peptides identified in this study have not been previously documented in the database, which provides an opportunity to enrich the BIOPEP-UWM library with new data on COX-2 enzyme inhibitors. The simulation results from BIOPEP-UWM also indicate that the peptides show different biological activities, including ACE, DPP-IV and α-glucosidase inhibition, suggesting their potential for various future applications. However, these results require further validation. The peptide toxicity predictions showed that none of the sequences were toxic, suggesting that the peptides produced during the gastrointestinal simulation of peanut worm collagen are safe to use.

**Table 2:** Peptide sequence of peanut worm collagen and its potential inhibitory effect

No.	Peptide sequence	*M*/Da	Toxicity prediction	Activity	Frequency of bioactive fragment (A)	Possible biological activity of protein fragment (B)	Peptide fragment	Accession number
1	ADIAGQAAQVLR	1212.37	Non-toxic	ACE inhibitor	2.89	0.00125173	IA, AA, AG, GQ, LR	A0A117DPD2
				DPP-IV inhibitor	4.05	0.00113499	IA, AA, AD, AG, QA, QV, VL	
				α-glucosidase inhibitor	1.16	0.00000324	AD, LR	
2	LNNEITTLR	1073.21	Non-toxic	ACE inhibitor	3.09	0.00086699	ITT, EI, LN, LR	A0A976XJT2
3	VGTVEK	631.35	Non-toxic	ACE inhibitor	4.63	0.00018075	VG, GT, VE, EK	A0A1A8HCI8
				DPP-IV inhibitor	4.63	0.00005181	EK, TV, VE, VG	
				α-glucosidase inhibitor	1.16	0.00000751	VE	

### Computational modelling for the binding structure of the peptide and COX-2

**Fig. 3:**
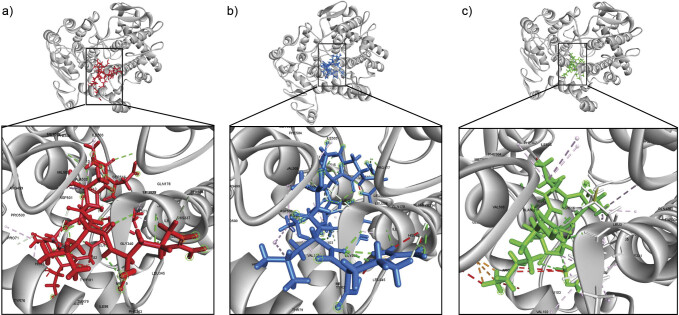
The interaction of peanut worm peptide and COX-2: a) ADIAGQAAQVLR, b) LNNEITTLR and c) VGTVEK. The grey ribbon was the visualization of COX-2, while red, blue and green were ADIAGQAAQVLR, LNNEITTLR and VGTVEK, respectively

The peptide sequences (ADIAGQAAQVLR, LNNEITTLR and VGTVEK) showed the most favourable binding conformations with COX-2, as shown in Fig. 3. Based on the binding energy values, VGTVEK (-4.41 kcal/mol) showed the strongest interaction, followed by ADIAGQAAQVLR (0.96 kcal/mol) and LNNEITTLR (1.28 kcal/mol). Binding energy values indicate the strength of the interaction between peptides (ligands) and COX-2 (receptors). A lower binding energy value means that a ligand requires less energy to bind or interact with its receptor.

Molecular docking showed that VGTVEK binds to the active site of COX-2 and forms hydrogen bonds with Gln178, Leu338, Ser339, Tyr371, Ile503, Phe504, Val509 and Ser516 and hydrophobic interactions with Met99, Val102, Val330, Ile331, Tyr334, Val335, Leu345, Trp373, Leu517 and Leu520. While ADIAGQAAQVLR shows interactions with Pro71, His75, Arg106, Gln178, Gln336, His337, Leu338, Ser339, Gly340, Tyr341, Tyr371, Met508, Val509, and Ala513, which form hydrogen bonds, and interactions with Ile331, Val335, Ala502, Phe504, and Leu520, which form hydrophobic interactions. LNNEITTLR, on the other hand, shows interactions with His75, Leu338, Arg499, Pro500, Asp501, Phe504, Gly512, Ala513 and Leu517, which form hydrogen bonds, and interactions with Val102, Val335, Tyr341, Leu345, Tyr371, Trp373, Met506 and Leu520, which form hydrophobic interactions. Additionally, all peptides exhibit several van der Waals forces with amino acid residues at the COX-2 active site and act competitively to inhibit COX-2. Their hydrogen bonding interactions with residues such as Leu338, Ser339, Arg499 and Phe504 in the active site mirror those of celecoxib, suggesting that they can selectively inhibit COX-2 (*48*).

The VGTVEK peptide proves to be a candidate with the highest COX-2 inhibitory activity. The interactions between celecoxib and the VGTVEK peptide are similar, as shown by hydrophobic interactions between the amino acid residues Val335 and Trp373, the hydrogen bonds with Leu338, Ser339 and Phe504, and the van der Waals contacts with Gly340, Phe367, Ala502 and Met508. As a result, it shows a more effective inhibition of the COX-2 enzyme. Chakrabarti and Wu (*49*) suggested that the Val residue at the N-terminus of the peptide contributes to its anti-inflammatory properties. Similarly, the VG segment of this peptide forms hydrogen bonds with Tyr371 and Ser516 in the active site of the enzyme, thereby inhibiting COX-2 activity, similar to the VGFP peptide from walnut hydrolysate (*47*). Several researchers also reported that the Lys position at the C-terminus of this peptide has a good anti-inflammatory effect (*50*). The position of the amino acids is crucial for the interactions between peptide and enzyme. Enzymes recognise and position different peptides in different ways and thus influence their binding and activity (*51*).

Inhibition of COX-2 by this peptide may help alleviate chronic inflammation, which is often associated with a number of degenerative diseases and autoimmune disorders (*52*). This peptide, which is derived from food, particularly collagen hydrolysate, has significant potential for the development of health-oriented functional diets and food additives. Moreover, this peptide has the potential to be used in foods for cancer-prevention due to its ability to suppress COX-2, a known carcinogen (*52*). In addition, these peptides offer a safer alternative to nonsteroidal anti-inflammatory drugs (NSAIDs), which are often associated with gastrointestinal and cardiovascular side effects (*53*). These peptides represent a safer long-term therapeutic alternative for chronic inflammatory conditions such as arthritis and autoimmune diseases, as their incorporation into natural anti-inflammatory treatments may minimise the risk of adverse effects (*53*). Although further research is necessary, the results suggest that peptides from *S. australe* collagen could serve as a valuable natural source for functional foods, nutraceuticals and pharmaceuticals with potent COX-2 inhibitory activity.

## CONCLUSIONS

This study presents peptides derived from peanut worm collagen as novel and promising natural inhibitors of COX-2, a key enzyme in inflammatory pathways. These peptides contribute to the growing number of marine bioactive compounds with anti-inflammatory properties. Three peptides (ADIAGQAAQVLR, LNNEITTLR and VGTVEK) were identified by *in vitro* digestion and molecular docking, with VGTVEK exhibiting the strongest COX-2 inhibition. These results emphasise the therapeutic potential of these peptides for use in functional foods, nutraceuticals and pharmaceuticals and offer new options for the treatment of inflammation.
